# SOCIAL ENGAGEMENT THROUGH VIDEO CHAT FOR OLDER INDIVIDUALS WITH AND WITHOUT COGNITIVE IMPAIRMENT

**DOI:** 10.1093/geroni/igz038.054

**Published:** 2019-11-08

**Authors:** Wendy Rogers, Qiong Nie, Lydia Nguyen, Raksha Mudar, Dillon Myers, Alan Gibson, Chantal Kerssens

**Affiliations:** 1 University of Illinois, Urbana-Champaign, Champaign, Illinois, United States; 2 University of Illinois, Champaign, Illinois, United States; 3 OneClick.Chat, Philadelphia, Pennsylvania, United States; 4 Eyiapp, Atlanta, Georgia, United States

## Abstract

Social engagement is a fundamental component of health and quality-of-life outcomes. However, there is a prevailing view that older adults primarily want to engage socially with current family and friends – that they are not interested in developing new relationships. That is an overgeneralization. We have found that older adults are interested in the opportunity to engage in social interactions with people who have shared interests. Technology can facilitate these interactions. We will describe our research with OneClick.chat, a web-based video chat system. We explored potential benefits of use by adults aged 70-85, including those with mild cognitive impairment (MCI), as well as barriers and facilitators to adoption. Participants saw value of this online social engagement platform and were able to use it with some initial training. They envisioned using OneClick not only for conversations but also for learning and doing activities with like-minded individuals.

